# Annotations

**Published:** 1900-10-27

**Authors:** 


### Annotations.

### The Harveian Oration.

The Harveian Oration was this year delivered by Professor Clifford Allbutt, F.R.S., who took for his theme a consideration of some of the difficulties with which Harvey had to contend, and especially those which arose from the movements of thought which had characterised the Middle Ages and had imposed fetters upon the minds of thinkers and investigators of every degree, not excepting Harvey himself. Thus even Harvey had to seek the motor of his circulating machine in the "quintessence"—the fifth element or source of energy, motion of the celestial spheres—and for Harvey also the heavenly bodies were still living and intelligent beings whose self-existent and perpetual motion was propagated down to sublunary matter. To recognise Harvey's greatness one must remember that he not only made the researches for which he is mostly remembered, but that he stood forth in medicine against humoralism, pneumatism, galenism, long-winded dialectics on critical days, coctions, derivatives, revulsives, and like abstractions, bequeathed by realism and uncritical subservience to texts; and that even these surroundings were projected against a still more lurid background of folk superstition, of astrology, alchemy, cheiromancy, cabalism, magic, vampires, and witch-burning. So much for Harvey. We listened to Professor Allbutt's words, and congratulated ourselves that now at last the centuries had gone round and that we had reached a point when we could appeal to facts and feel confident that our conclusions would be judged in the light of reason by an intelligent public freed from all mysticism and tomfoolery. Alas! Alas! Even while Professor Allbutt, as the representative of all that is modern, was lifting up his voice and by implication thanking Heaven that this century was not as other centuries, nor even as that poor century two hundred years ago when magic and superstition ruled men's minds—even while this was going on at one side of Trafalgar Square, just over the way people were flocking to listen to an apostle of Christian Science! It is too soon to talk of the emancipation of the human mind or the progress of the ages. There is no story too wild, no theory too extravagant to be accepted of the people, while even in medicine dogma is constantly lifting up its head just as of old. Perhaps now more than ever is the teaching of Harvey required to keep us in the straight path.

### American "Political Hospitals."

The national sentiment is strong among Americans, and the outside critic of their institutions is not loved; in fact, he is not unseldom accused of partiality, and, above all, of ignorance. But when outsiders find Americans speaking about their own affairs he may perhaps, without offence, draw his own conclusions, and from the remarks made by Dr. Joseph Price,[1] of Philadelphia, at the annual meeting of the American Association of Obstetricians and Gynæcologists the conclusion seems inevitable that the "political hospital," as it exists in America, is not an altogether desirable institution. The object of his remarks appeared to be to prove the advantages of well-organised private hospitals. He said that such hospitals gave the operator the best opportunity for doing good work. In well-managed private institutions the patient had one or more attendants with well-regulated relays, while, on the other hand, "in public institutions the speaker rarely found a nurse in large wards, the only attendants about the patients being a convalescent patient or an old pelican of an attendant." We quite agree that a private institution, if well managed, may be made very attractive, but we should hardly have dared to speak so disparagingly of the public institutions of so great a country as Mr. Joseph Price has done. We wonder whether he is right, and whether what he calls political, i.e., rate-supported hospitals, are so badly managed as he suggests. The matter is one of considerable interest just now, when various people are gravely suggesting that the best way out of all hospital difficulties would be to throw them on the rates, and take for our model the modes of hospital management now current in America.

[1] Medical Record, Oct. 6.

### The Signs and Symptoms of Plague.

We have received from the medical officer of the London County Council an admirable résumé of the signs and symptoms of plague, drawn up by Mr. James Cantlie, together with a notification stating that as few medical men have had the opportunity of seeing cases of plague, the Council have engaged the services of Mr. Cantlie to visit any person in London suspected to be suffering from that disease with a view to assisting in the diagnosis, and that any medical practitioner desiring to avail himself of these services should apply at the office of the Public Health Department, 8 St. Martin's Place, W.C.

Mr. Cantlie says that although there are certain signs, symptoms, and post-mortem appearances which are common to all varieties of plague, one or another clinical symptom is apt to predominate in each case. The disease may therefore be described as occurring in the following forms or types:—(1) bubonic; (2) septicæmic; (3) pneumonic; (4) the nervous type; (5) the toxic or fulminant type; (6) the puerperal type—cases in which the disease leads to abortion or miscarriage; (7) pestis ambulans, a mild type (most commonly met with in children), but one which is very dangerous so far as the dissemination of infection is concerned; (8) pestis minor, which is described as an abortive type of plague ending in a chronic adenitis, often with suppuration.

Now, so far as diagnosis is concerned, proof that a person is suffering from plague can only be arrived at by proving the presence of the plague bacillus. But "the sudden onset, the marked prostration, the mental aberration, the splitting headache, the rise in temperature, the furred tongue, when taken in conjunction with tenderness and pain in some one of the groups of glands, are sufficient to indicate the necessity for a speedy microscopic search for the plague bacillus." In practice it will probably be found that the chief difficulty will arise in regard to the pneumonic and the puerperal type. The bubonic type will in the end declare itself by the buboes, and in the septicæmic, the fulminant, and the nervous type, the symptoms at least will be such as to show that something strange and something demanding further investigation is going on. But pneumonia shows itself in so many types, and abortion may be produced by so many causes, and in the homes of the poor it so commonly leads to serious fever, that unless a man has his eyes open to the possibility of plague the presence of pneumonia or the occurrence of an abortion may easily be taken as explaining the whole case and rendering further inquiry unnecessary. What are really the most infectious forms of the disease may thus be overlooked, as was very markedly the case in the earlier days of the outbreak in Bombay.

